# Effective removal of Pb(II) ions using piperazine-modified magnetic graphene oxide nanocomposite; optimization by response surface methodology

**DOI:** 10.1038/s41598-022-13959-8

**Published:** 2022-06-10

**Authors:** Mousa Alboghbeish, Arash Larki, Seyyed Jafar Saghanezhad

**Affiliations:** 1 Department of Marine Chemistry, Faculty of Marine Science, Khorramshahr University of Marine Science and Technology, Khorramshahr, Iran; 2 ACECR-Production Technology Research Institute, Ahvaz, Iran

**Keywords:** Chemistry, Nanoscience and technology

## Abstract

In this research, the piperazine-modified magnetic graphene oxide (Pip@MGO) nanocomposite was synthesized and utilized as a nano-adsorbent for the removal of Pb(II) ions from environmental water and wastewater samples. The physicochemical properties of Pip@MGO nanocomposite was characterized by X-ray diffraction analysis (XRD), Field emission scanning electron microscopy (FESEM), Transmission electron microscopy (TEM), Energy-dispersive X-ray spectroscopy (EDAX), Thermo-gravimetric analysis (TGA), Vibrating Sample Magnetometery (VSM) and Fourier-transform infrared spectroscopy (FT-IR) analysis. In this method, the batch removal process were designed by response surface methodology (RSM) based on a central composite design (CCD) model. The results indicated that the highest efficiency of Pb(II) removal was obtained from the quadratic model under optimum conditions of prominent parameters (initial pH 6.0, adsorbent dosage 7 mg, initial concentration of lead 15 mg L^−1^ and contact time 27.5 min). Adsorption data showed that lead ions uptake on Pip@MGO nanocomposite followed the Langmuir isotherm model equation and pseudo-second order kinetic model. High adsorption capacity (558.2 mg g^−1^) and easy magnetic separation capability showed that the synthesized Pip@MGO nanocomposite has great potential for the removal of Pb(II) ions from contaminated wastewaters.

## Introduction

Disposal of industrial effluents and wastewaters is considered as one of the most important challenges in the industrial world today. Due to the toxic ingredients of these effluents such as, heavy metal ions and dyes, they are the main cause of pollution of rivers, lakes and underground waters^1,2^. Unlike organic pollutants, heavy metal ions are not only degradable or decomposable but also can accumulate in biotic and abiotic systems through the food chain, drinking water and air, resulting in serious damage to the environment and human safety^3^. The most notorious heavy metals that cause significant environmental pollution are lead, chromium, mercury, cadmium, arsenic, zinc, copper, and nickel. The concentration of some of them have reached dangerous levels both for the environment and humans. Accordingly, reduction of such pollutants is one of the most significant steps in wastewater treatment^4^. Among the various toxic metal ions, lead is a highly toxic pollutant that is released into the environment due to industrial activities, including mining, plating, battery production, metal smelting, oil refining, printing, and so on. Accumulation of Pb(II) ions in the human body lead to various health consequences, such as: anorexia, gastrointestinal colic, anemia, neurasthenia, kidney and liver damage, and even cancer^5^. Therefore, in order to environmental clean-up, it is absolutely essential to design appropriate technologies and prepare effective materials for complete removal or reduction of Pb^2+^ ions to an acceptable level, before discharge^6^. It should be notified that careful sensing of pollutants and chemicals are also beneficial^7^.

There are many traditional methods for removing lead, including ion exchange, chemical precipitation, electrodeposition, membrane filtration and reverse osmosis^8^. However, these techniques usually suffer from some limitations including complexity and high cost of their operation, potential secondary pollution, difficulty in recycling and poor efficiency in low concentration of lead^9,10^. Among the various treatment technologies, adsorption is currently preferred as a non-hazardous method for the removal of heavy metal ions due to its cheapness, selectivity, high efficiency, simple processes, reusability, flexibility in design and availability of different adsorbents^11^. Generally, an ideal adsorbent should have a high surface area and also adsorption sites, so that the adsorption process takes place in a short equilibrium time^11,12^. In the last two decades, by the development of novel nanotechnologies and the advent of nanomaterials^13,14^, scientists have been attracted to this field and various novel adsorbents have been emerged^15^. Due to various advantageous in which appears by reducing the size of the adsorbent to nanometers and increasing the surface area and thus increasing the active sites, the adsorption capacity of these materials increases significantly^16,17^.

Quite recently some novel strategies have been proposed for lead removal or adsorption via nanomaterials. In this regard, removal of lead from aqueous solutions using three biosorbents was evaluated by Rezaei et al., which the best adsorption capability was scales of *Rutilus kutum* and *Oncorhynchus mykiss* and the shells of *Cerastoderma glaucum* in descending order^18^. Furthermore, antimicrobial nanocomposite adsorbent based on poly(meta‑phenylenediamine) has been proposed for lead(II) removal from aqueous solutions^19^. Ahadi et al., have also proposed MIL‑53(Al) as a Metal–Organic Framework for separating lead ions from aqueous solutions^20^. Wang et al., presented a rapid removal method for Pb(II) from aqueous solution using branched polyethylenimine enhanced magnetic carboxymethyl chitosan^21^. Hu et al. had also prepared magnetic, water-soluble hyperbranched polyol functionalized graphene oxide nanocomposite which was utilized for the removal of synthetic dyes and also Pb(II) ions^22^. Wang et al. have also utilized ultrasound-assisted xanthation of alkali cellulose optimized by RSM for Pb(II) sorption^23^. This research group has conducted Pb(II) sorption from aqueous solution by novel biochar loaded nanoparticles^24^. They have also utilized carboxyl functionalized *Cinnamomum camphora* for the biosorption of Cd(II), Cu(II) and Ni(II)^25^.

Recently, graphene oxide (GO) as a single-layered two-dimensional (2D) nanomaterial has aroused great interest among analytical chemists due to large surface area, high mobility and good conductivity. GO has been extensively used as an adsorbent in solid phase extraction of various organic and inorganic contaminants^26^. However, the dispersion of GO nanosheets is very high and their separation from the solution medium is very difficult and also time consuming. In order to facilitate separation after the adsorption process, the creation of magnetic properties through the fabrication of magnetic graphene nanocomposites is recommended^26–30^. Generally, Fe_3_O_4_ magnetic nanoparticles have been widely used in the construction of nanocomposite adsorbents in magnetic SPE techniques due to their simplicity in synthesis and ease of application^31–34^, but pure Fe_3_O_4_ nanoparticles are rapidly oxidized in the atmosphere and are not suitable for efficient adsorption in complicated matrices. In order to overcome these limitations and enhance the applicability of these types of adsorbents in real wastewater, the Fe_3_O_4_ surface must be functionalized by a modifier with appropriate functional groups^8,35^. Hence, by designing a solid hybrid of magnetic graphene oxide (MGO) with suitable modifier, a good adsorbent can be prepared to remove pollutants.

Due to the various benefits of functionalization of nanomaterials, chemists have tried to covalently or noncovalently functionalize graphene oxide with various chemicals^36^ and also biochemicals^37^, so that they are endowed with unique characteristics and excellent abilities^38^. In recent years piperazine functionalization has been performed for some applications including heavy metal ion removal^39^, loading of 2-mercaptobenzothiazole^40^, acid recovery by diffusion dialysis^41^ and catalysis^42^.

Along with our recent research interests in preparing novel adsorbents^17,43^ we decided to take advantage of the coordinating capability of piperazine for metal ions and also the high surface area of grapheme oxide. Thus it was decided to investigate the capability of piperazine-functionalized magnetic graphene oxide (Pip@MGO) nanocomposite for the removal of Pb(II) from the aqueous environment. The fabricated Pip@MGO nanocomposite was characterized by XRD, FESEM, TEM, EDAX, TGA, VSM and FT-IR analysis. The most significant parameters in the removal efficiency, including solution pH, initial lead concentration, adsorbent dosage, and contact time, were considered and the optimal values of these variables were evaluated by a statistical approach using response surface methodology (RSM) based on a central composite design (CCD) model. In addition, the isotherm modelling and kinetics parameters were studied to understand the mechanism of adsorption of Pb^2+^ ions with Pip@MGO adsorbent.

## Experimental

### Reagents

All reagents and chemicals used in this work were of analytical grade, without further purification, and ultrapure distilled water was used in the experiments. The chemicals including, graphite powder, iron(III) chloride hexahydrate, iron(II) chloride tetrahydrate, 3-chloropropyltriethoxysilane, piperazine anhydrous, potassium permanganate, hydrogen peroxide (30%), sulfuric acid (98%), hydrochloric acid (37%), sodium hydroxide, toluene and ethanol were purchased (Merck, Darmstadt, Germany). A stock solution of 1000 mg L^−1^ of Pb^2+^ was prepared by dissolving 0.159 g of Pb(NO_3_)_2_ (Merck, Darmstadt, Germany) in 10 mL of concentrated HNO_3_ and diluted to 100 mL in a volumetric flask. The working solutions were prepared daily with suitable dilution of this stock solution. The solutions of nitric acid and sodium hydroxide (in the concentration range of 0.01–1.0 M) were used to adjust the pH.

### Characterizations

A flame atomic absorption spectrometer from GBC Company (Sidney, Australia, Savanta Model) equipped with a deuterium lamp and hollow-cathode lamp was used for determination of lead. Morphology of the synthesized nanocomposite adsorbent was characterized with a by field emission scanning electron microscopy (FESEM, TESCAN, MIRA III model, Czech Republic) instrument. In addition, EDAX and element mapping analysis (EMA) for the surface components of the samples were analysed using EDAX-MAP (FESEM, TESCAN, MIRA III model, Czech Republic). The structure and size of prepared Pip@MGO was investigated by a transmission electron microscope (JEM-1011 model, Japan TEM, Zeiss-EM10C-80 kV). FT-IR spectra was recorded with a Fourier Transform Infrared spectrometer (Thermo Nicolet, AVATAR model, USA) at room temperature in KBr pellets. Magnetic properties of produced nanocomposite were obtained by a vibrating sample magnetometer (VSM, MDKB model, Meghnatis Daghigh Kavir Co., Kashan, Iran). Thermogravimetric Analysis (TGA) was done using a TA Instruments analyzer (Q600, USA) by scanning to 800 °C with a heating rate of 10 °C min^−1^. An ultrasonic water bath (ALEX, 8 L, power 170 W and frequency 32 kHz) was applied to disperse the of Pip@MGO nanocomposite adsorbent in the aqueous solutions. The pH adjustment of sample solutions were done with a Metrohm digital pH meter (632 model, Switzerland, Swiss) with a combined glass electrode.

### Synthesis of Pip@MGO nanocomposite

#### Preparation of graphene oxide (GO)

Graphene oxide (GO) was prepared according to Hummers' method with some modifications. Accordingly, 1.0 g of graphite powder was added to 50 mL of H_2_SO_4_ (98%) in an ice bath. Afterwards, KMnO_4_ (2 g) was added slowly. The addition should be in a way that the temperature does not exceed abruptly. After 2 h of stirring below 10 °C, and 1 h at 35 °C; 50 mL of deionized water was added. The mixture was heated at 85 °C for 1 h. Then 10 mL of H_2_O_2_ (30%) was added in which the solution turned bright yellow. The mixture was filtered and was washed with HCl (5%) and deionized water for several times. Finally the obtained GO was dried at 60 °C in oven for 24 h^44^.

#### Preparation of magnetic graphene oxide (MGO)

In a 250 mL round-bottom flask, 0.5 g of GO was added to deionized water (100 mL) and it was sonicated for 15 min. Subsequently, in another flask, FeCl_3_·6H_2_O (1 g) and FeCl_2_·4H_2_O (0.4 g) were mixed totally in deionized water (50 mL), for 1 h at 80 °C. The flask containing GO was added to the solution of Fe(III)/Fe(II) and then sonicated for 30 min in sonication bath. The mixture was heated to 80 °C and after 30 min, 15 mL of ammonia (30%) was added to it, resulted in MGO. The mixture was stirred for another 30 min and MGO was separated with an external magnet. The MGO was washed several times with deionized water and finally the obtained MGO was dried at 60 °C in oven for 24 h.

#### Preparation of piperazine modified magnetic graphene oxide (Pip@MGO)

**Figure 1:**
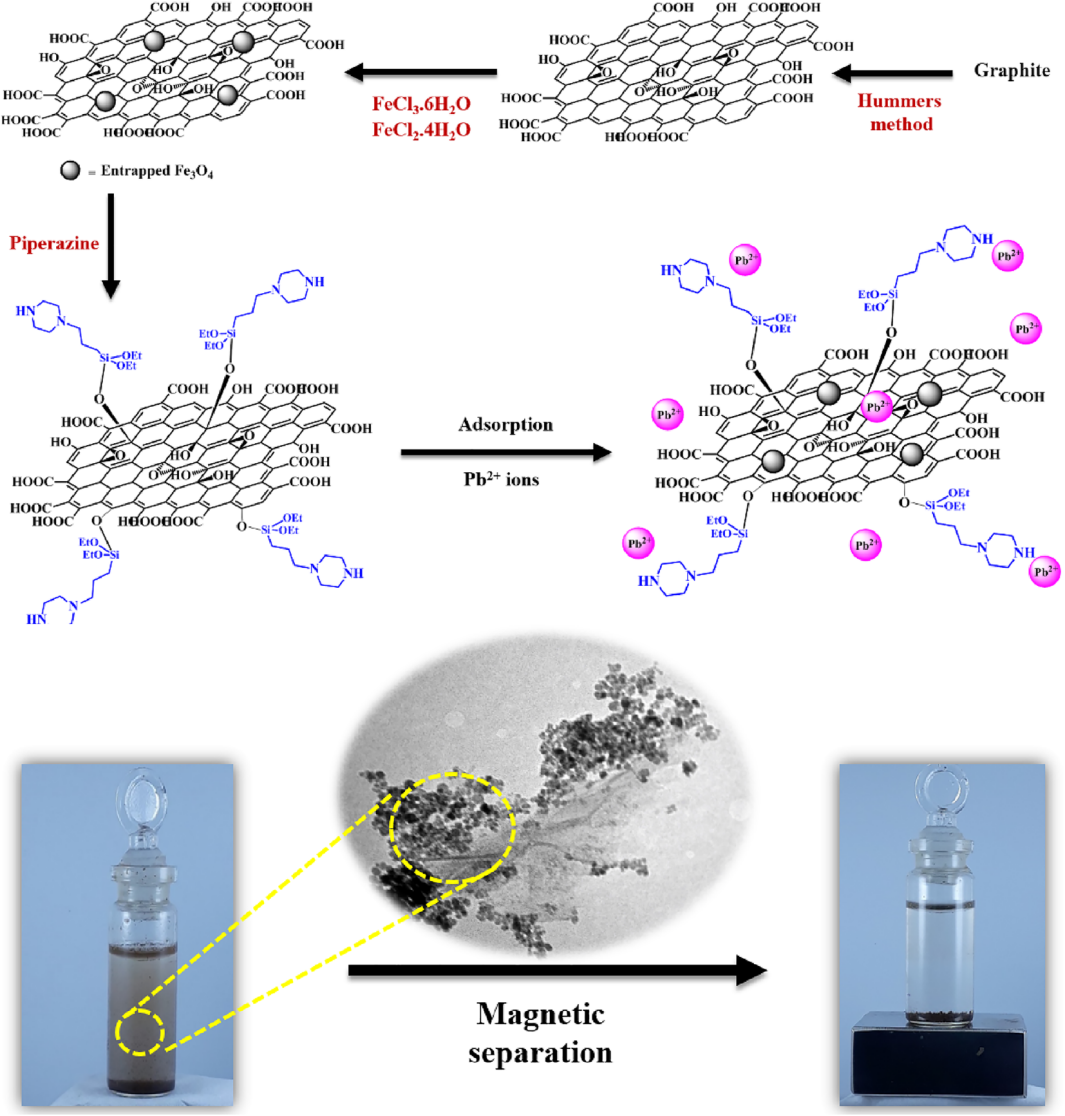
Schematic representation of synthesis of Pip@MGO nanocomposite and postulated mechanism for Pb^2+^ ions adsorption.

In a 100 mL round bottom flask, 0.5 g of MGO was added to 50 mL of toluene and the mixture was sonicated for 15 min. In another flask, 3-chloropropyltriethoxysilane (1 mmol, 0.24 g) and piperazine (1 mmol, 0.09 g) was premixed in 20 mL of toluene for 1 h at room-temperature. The latter flask was added to MGO containing flask and the mixture was refluxed for 24 h. Afterwards, Pip@MGO was separated with an external magnet and it was washed several times with ethanol. Finally, the collected Pip@MGO nanocomposite was dried at 60 °C in oven for 24 h and stored. A schematic representation of synthesis of Pip@MGO nanocomposite and its magnetic separation in adsorption processes was illustrated in Fig. 1.

### Collection of real samples

Four water samples including river water of Arvand Rud and Bahmanshir (Khuzestan Province, southwest of Iran), Persian Gulf seawater (Mahshahr, Khuzestan Province, Iran) and petrochemical wastewater sample (Abadan Petrochemical Company, Khuzestan Province, Iran) were collected in amber glass containers, which were previously pre-cleaned and acid-washed, and filtered through filter paper (Whatman, No. 1 Quantitative Filter Papers, 110 mm). Then, the treated samples were stored at 4 °C in the dark until analysis.

### Adsorption tests

In order to adsorb Pb(II) ions on the Pip@MGO nanocomposite, a batch method was used. According to response surface methodology, 30 experiments were done and the influence of effective parameters, including solution pH, initial concentration of Pb(II), adsorbent dosage and contact time were investigated on the lead removal efficiency (Supplementary Table S1). Briefly, 10 mL of solution containing different concentrations of lead at a given pH (in the range of 5–7) was transferred to the test tubes and the known weight of adsorbent was added and sonicated in an ultrasonic bath for 2 min, then shacked (at 200 rpm) for a certain period of time, using an incubator shaker. After contact time, the liquid and solid phases were separated by an external magnet and the residual Pb^2+^ concentration was measured by FAAS. The removal percentage (% R) and adsorption capacity of Pb(II) ions (q_e_; mg g^−1^) were determined by the Eqs. (1) and (2):

1 $$\% R=\frac{{C}_{0}-{C}_{e}}{{C}_{0}}\times 100,$$

2 $${q}_{e}=\frac{\left({C}_{0}-{C}_{e}\right) V}{M},$$

where C_o_ is initial and C_e_ is the final concentration of Pb(II) ions (mg L^−1^), V (L) is the volume of solution and M (mg) is the mass of adsorbent.

### Experimental design

Response surface methodology (RSM) is a well-known and useful method that is widely used to optimize adsorption techniques and experimental design, modeling in chemical reactions and industrial processes^45^. In this work, the central composite design (CCD) under RSM was applied for designing the experiments. Therefore, the effect of four independent variables, including pH (in the range of 5–7), initial concentration of lead (in the range of 5–25 mg L^−1^), adsorbent dosage (in the range of 1–13 mg) and contact time (in the range of 2.5–52.5 min) on the Pb(II) removal efficiency (% R) were investigated. The statistical software Design Expert (Version 11.0.3.0), Stat-Ease, Inc. was applied to analyze the experimental data. As shown in Table 1, for four independent factors at 5 levels (− α, − 1, 0, 1, + α) thirty runs were designed, including six repeated runs for a central point, eight axial runs and 16 full factorial runs (Supplementary Table S1). In this study, the proposed model was determined and confirmed by analysis of variance (ANOVA). In addition, R^2^, predicted R^2^, adjusted R^2^ and F-test values were performed to evaluate and express the quality of the produced models.

**Table 1:** Levels of the independent variables for CCD experiments.

Variables	Units	Type	Coded levels				
			− α	− 1	0	+ 1	+ α
Initial pH	–	A	5	6	7	8	9
Lead ions conc	(mg L^−1^)	B	5	10	15	20	25
Adsorbent dosage	(mg)	C	1	4	7	10	13
Time	(min)	D	2.5	15	27.5	40	52.5

## Results and discussion

### Characterization studies

**Figure 2:**
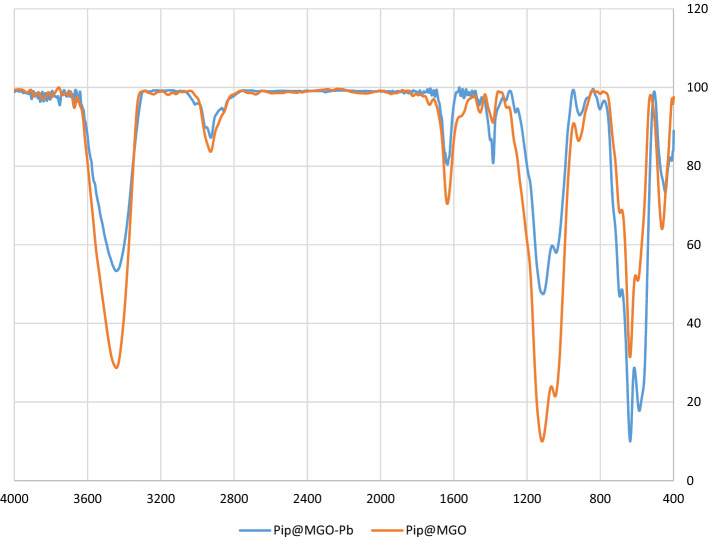
FT-IR spectrum of Pip@MGO and Pip@MGO-Pb nanocomposite.

In the FT-IR spectrum of the Pip@MGO (Fig. 2), the stretching vibration of C-H bonds of the graphene oxide (GO) is apparent in the 2928 cm^−1^^46^. The hydroxyl groups and also carboxylic groups on the surface of the GO is obvious at 3443 cm^−1^. It should be mentioned that the Fe–O vibration is also seen at 580 cm^−1^. The characteristic peaks located at 1635 cm^−1^ could be assigned to the bending vibration and stretching of –OH from the water molecules and structural hydroxyl groups^47^. The CH_2_ bending vibration is located at 1456 cm^−1^ which can be attributed to the methylene groups of piperazine and also the propyl silane linker. The C=O stretching vibration of COOH groups on GO is observed at 1727 cm^−1^^48^. The Si–O–Si vibration of the siloxane appear about 1040 and 1117 cm^−1^^49^. The FT-IR spectrum of Pb-Adsorbed Pip@MGO (Pip@MGO-Pb) was also recorded (Fig. 2).

**Figure 3:**
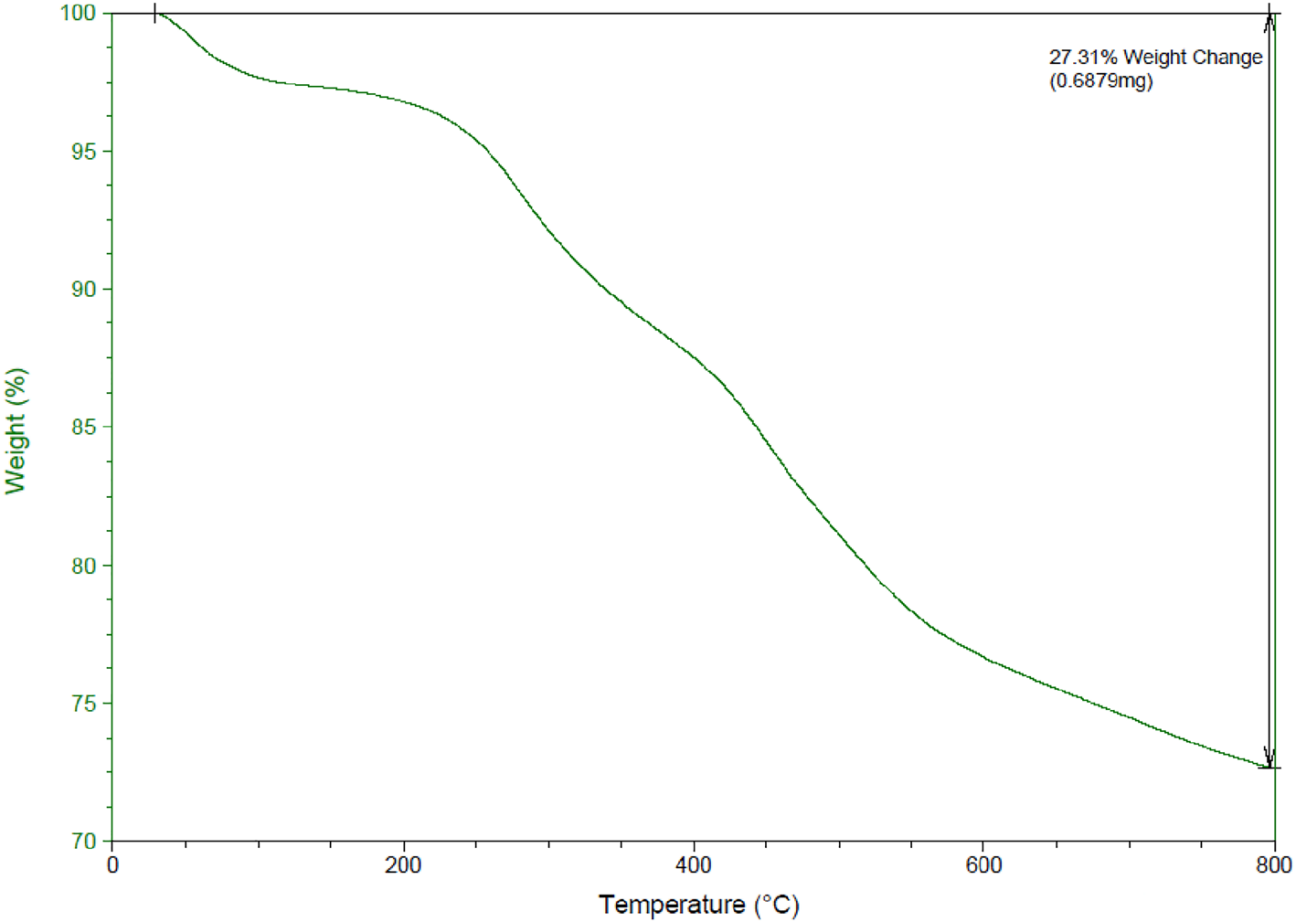
TGA diagram of Pip@MGO nanocomposite.

**Figure 4:**
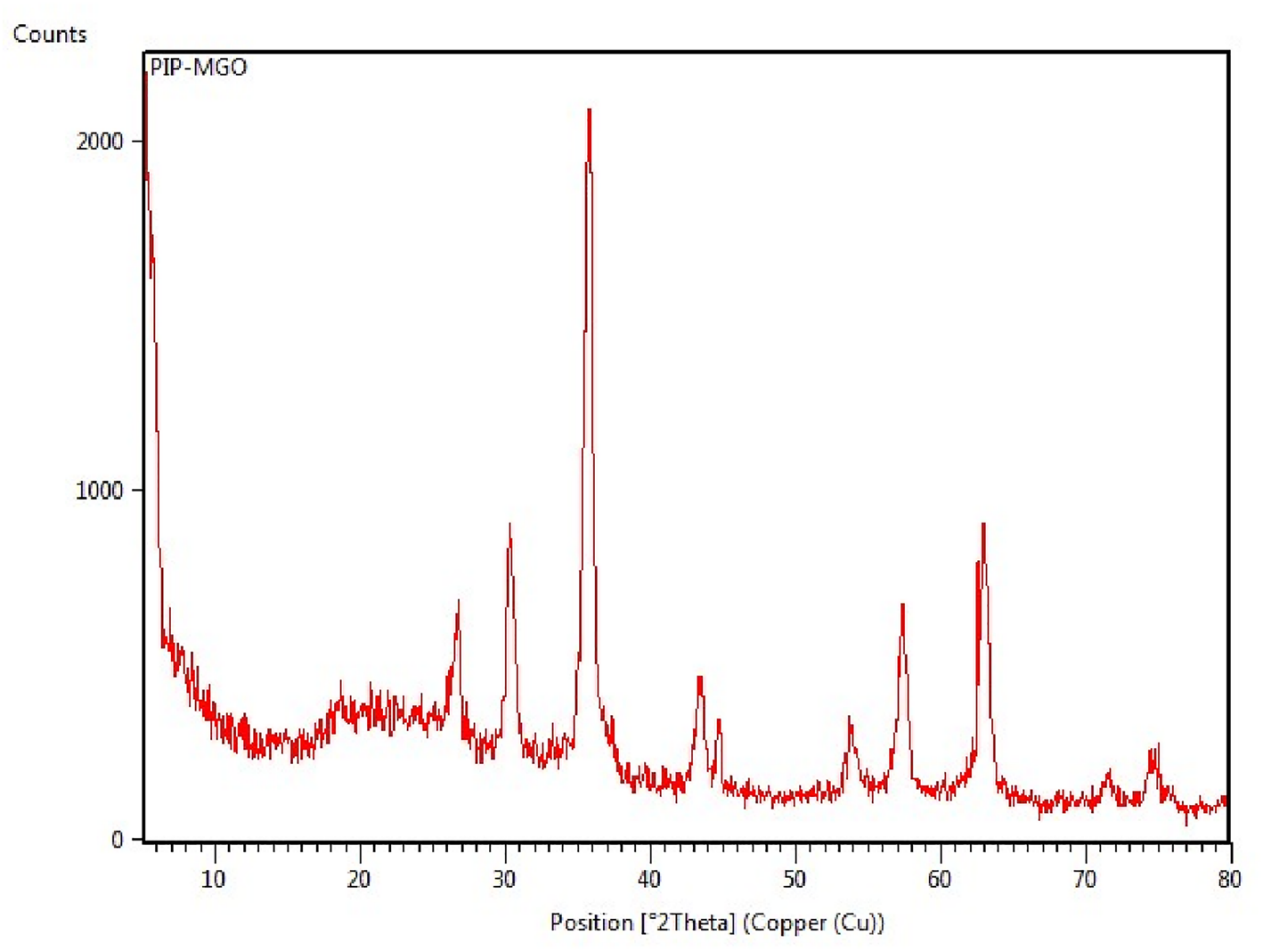
XRD pattern of Pip@MGO nanocomposite.

Thermogravimetric analysis (TGA) diagram of Pip@MGO is presented in Fig. 3. The first weight loss from room-temperature to 250 °C is due to the removal of adsorbed moisture entrapped physically in the nanocomposite. The second weight loss occurs from 250 to 800 °C which can be attributed to the breakdown of organic moieties from the nanocomposite. According to Fig. 4, the XRD pattern of the nanocomposite clearly shows the characteristic 2θ peaks of Fe_3_O_4_ at 30.2°, 35.52°, 43.5°, 54, 57° and 63° are attributed to the crystal planes of magnetite at 220, 311, 400, 422, 511 and 440, respectively^50^.

**Figure 5:**
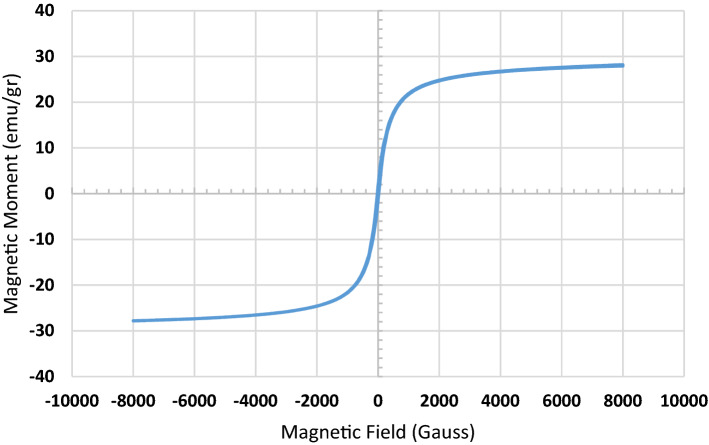
VSM curve of Pip@MGO nanocomposite.

The VSM curve of produced adsorbent is presented in Fig. 5. The results demonstrate that the nanocomposite contains magnetite nanoparticles which are super paramagnetic and the highest saturation magnetization is at 27.9 emu/g. It is apparent from the magnetization that the nanocomposite is sufficiently magnetic to be easily separable via an external magnet.

**Figure 6:**
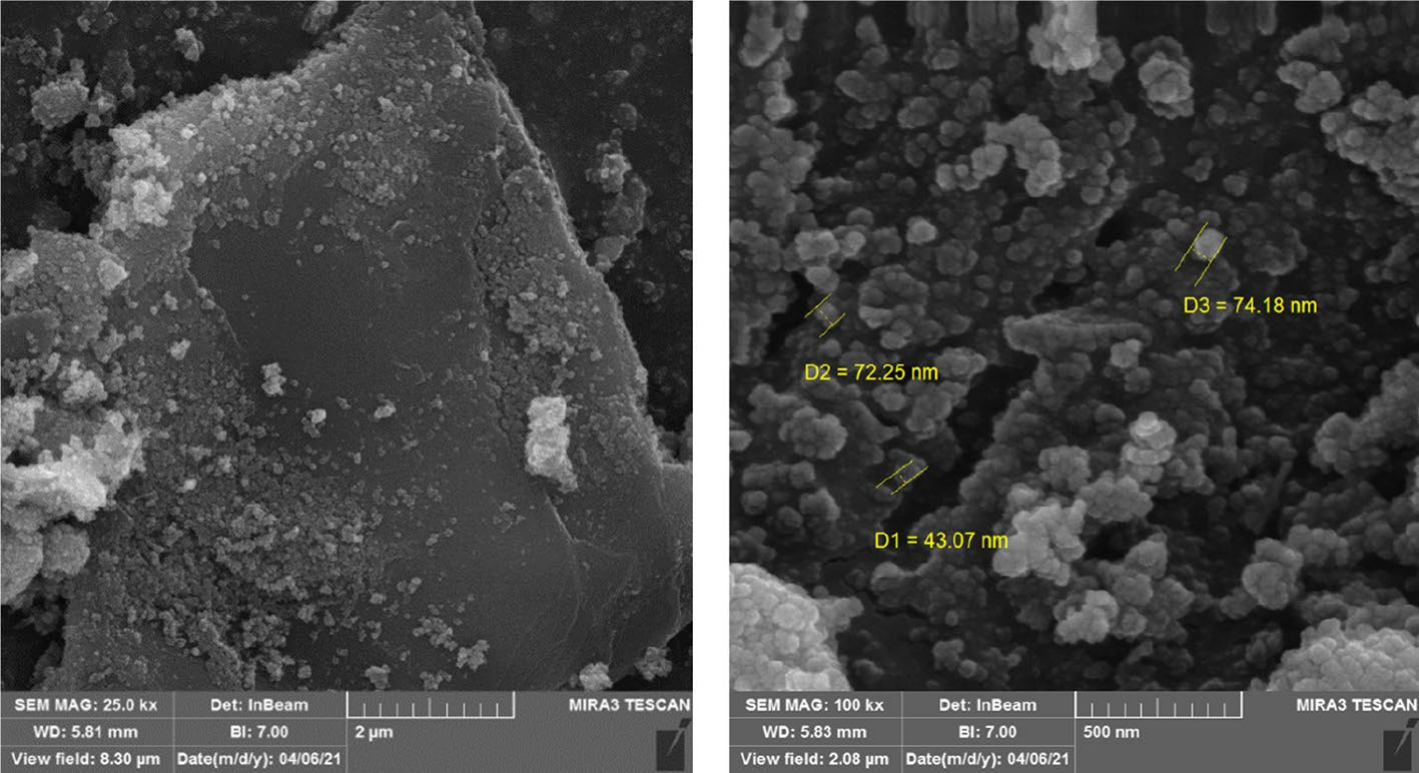
SEM images of Pip@MGO nanocomposite.

**Figure 7:**
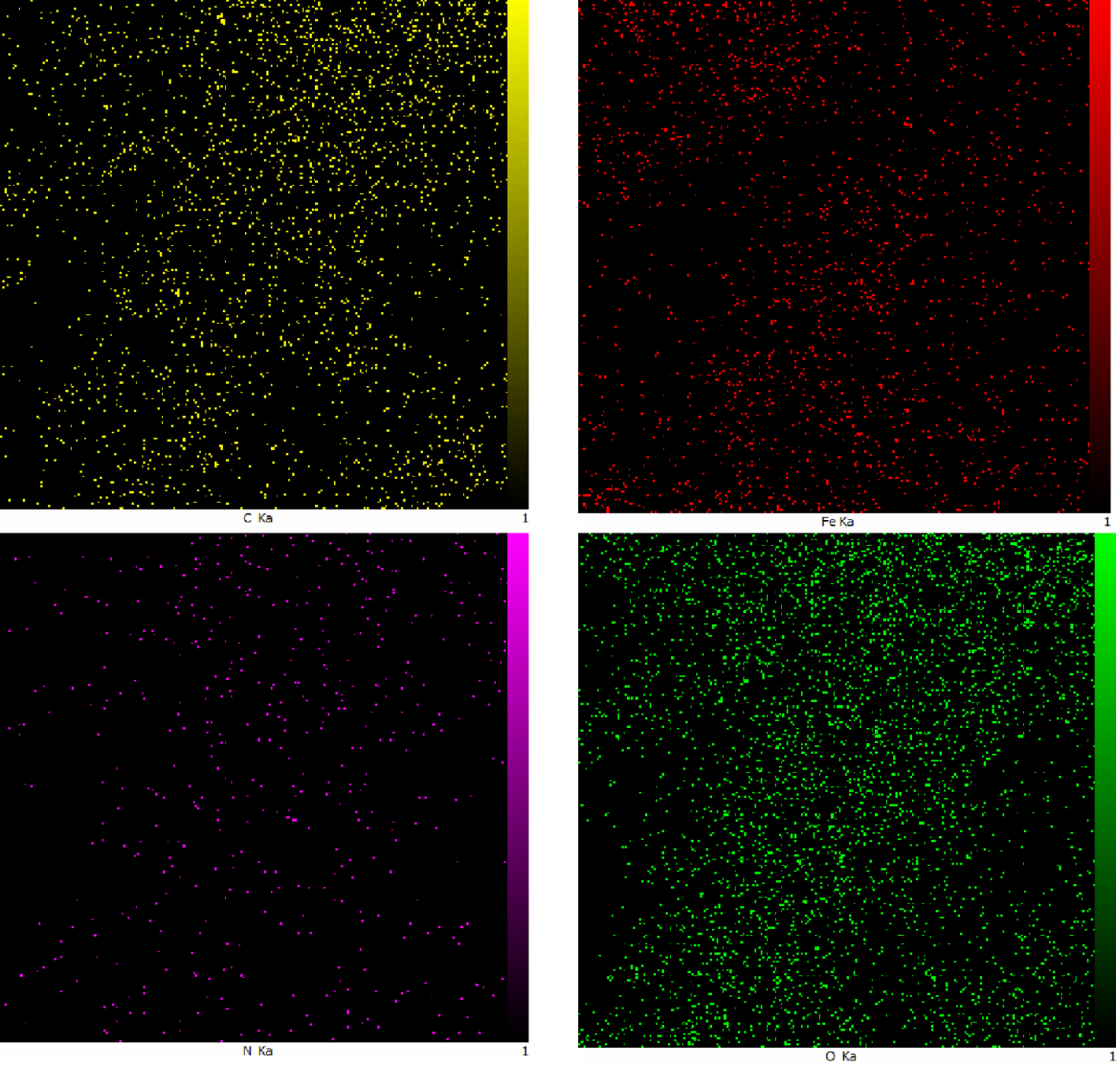
EDAX Mapping of Pip@MGO nanocomposite.

**Figure 8:**
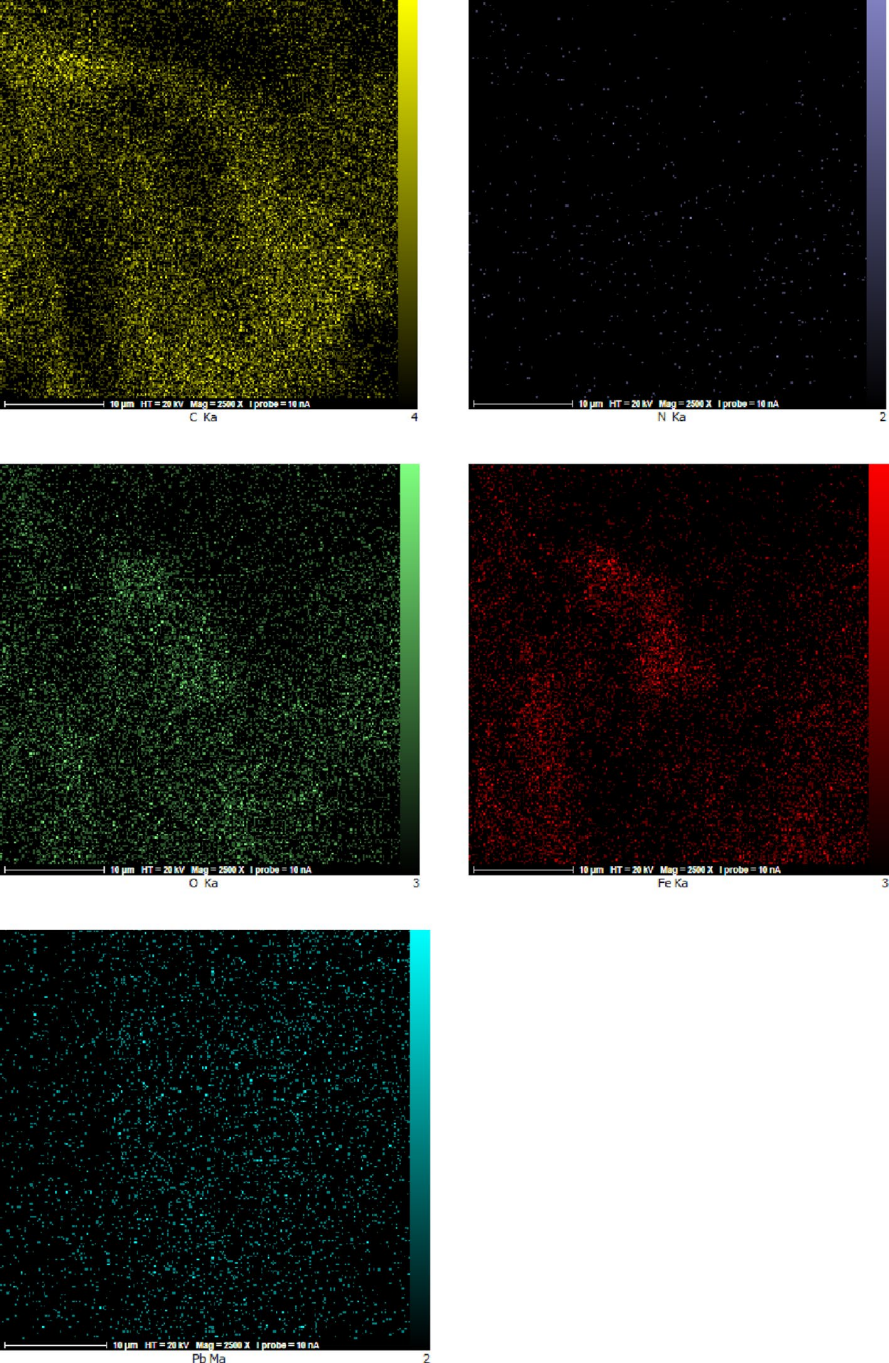
EDAX Mapping of elements in Pb^2+^ ions adsorbed Pip@MGO nanocomposite.

**Figure 9:**
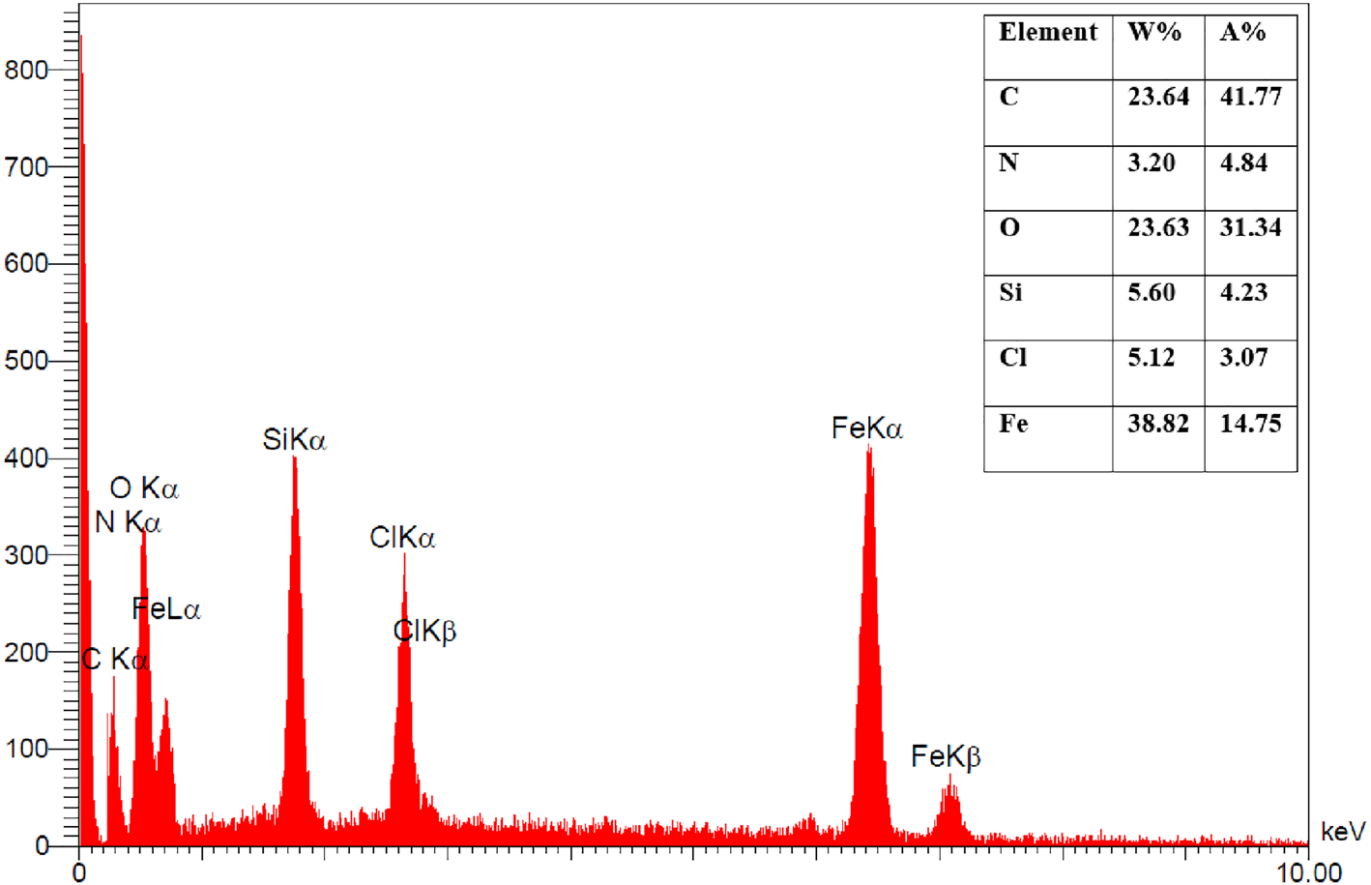
EDAX analysis of Pip@MGO nanocomposite.

**Figure 10:**
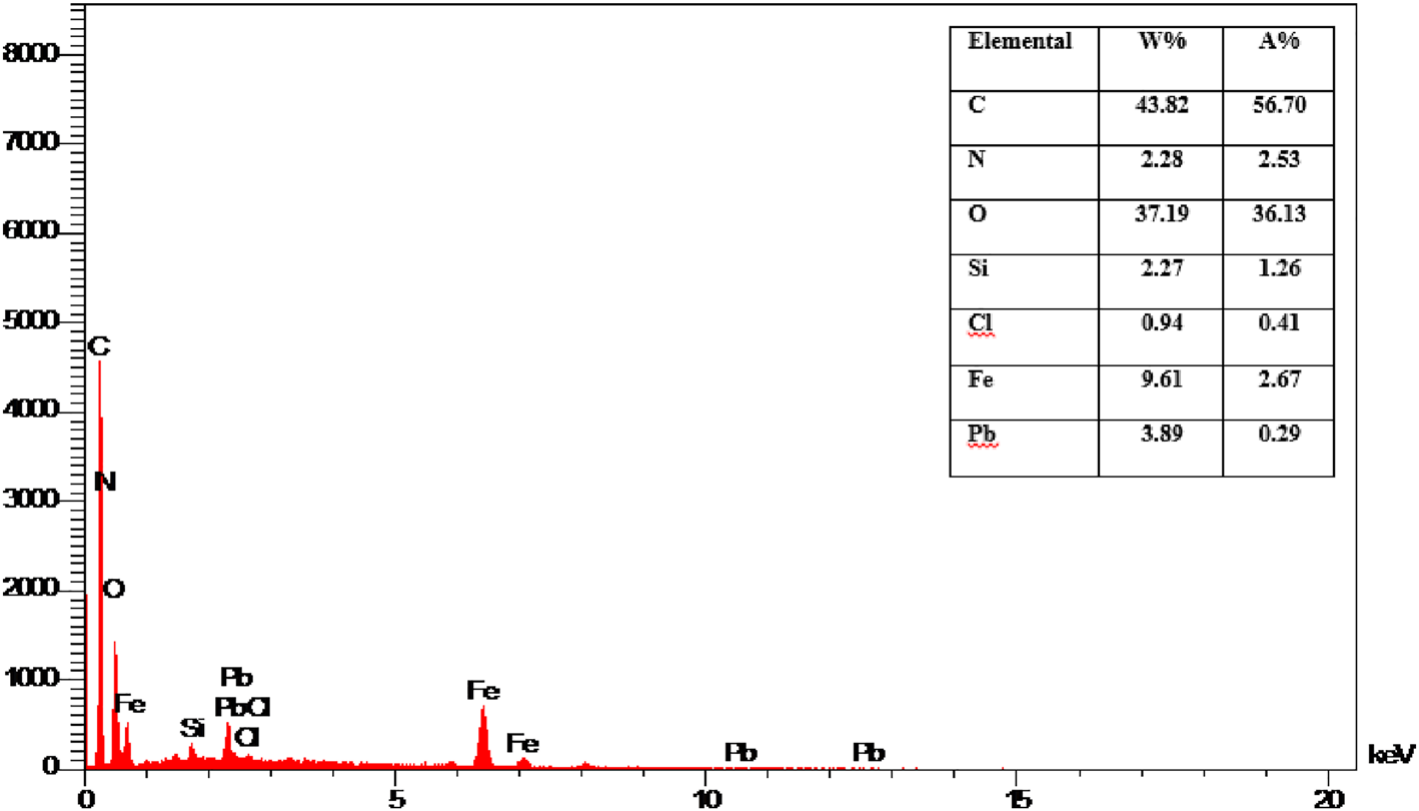
EDAX analysis of Pb^2+^ adsorbed Pip@MGO nanocomposite.

The SEM images (Fig. 6) of the nanocomposite at two different magnifications has been recorded. According to SEM images, the layered character of the graphene oxide and also the magnetite nanoparticles are easily observed. The nanoparticles are agglomerated to some extent, but they have been distributed on the whole surface of the graphene oxide. The distribution of elements in the specified area by EDAX mapping has been shown in Fig. 7. It can be seen that nitrogen as a constituent of the piperazine group has been distributed uniformly all over the specific area which is an indication of the even functionalization of the whole surface without any accumulation. Furthermore the EDAX mapping of Pip@MGO-Pb was also recorded (Fig. 8). Figure 9 illustrates the results of energy-dispersive X-ray spectrum (EDAX) analysis of Pip@MGO. This data indicates the approximate chemical composition and confirms successful immobilization of piperazine which contains nitrogen. The iron is also present in the analysis due to the presence of Fe_3_O_4_ nanoparticles in the nanocomposite. The results of energy-dispersive X-ray spectrum (EDAX) analysis of Pip@MGO-Pb is also presented in Fig. 10.

**Figure 11:**
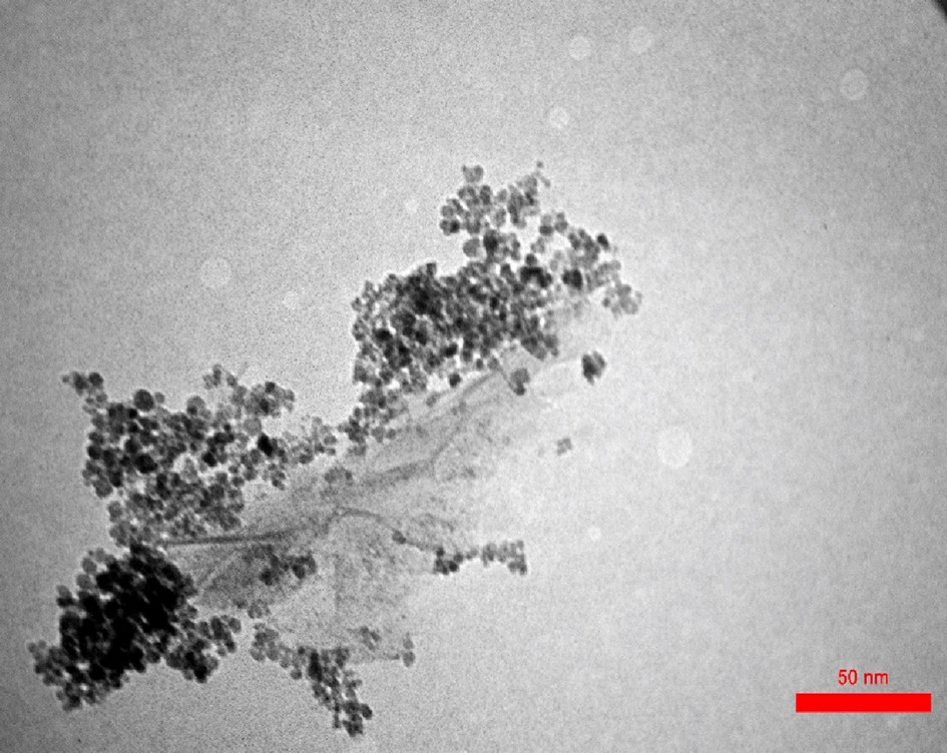
TEM image of the Pip@MGO.

TEM images of the Pip@MGO nanocomposite is illustrated in Fig. 11. The layered 2D character of the graphene oxide is beautifully viewed in the TEM image which contains the magnetite nanoparticles located on its surface.

### Suggested mechanisms of adsorption

In order to examine the mechanism of adsorption, the FT-IR and EDAX analysis of the lead (II)-adsorbed nanocomposite was recorded. From the general aspect, it can be concluded that the lead (II) ions are attracted toward free nitrogens of piperazine and also hydroxyl groups of graphene oxide (GO). According to FT-IR (Fig. 2) of Pb-adsorbed nanocomposite, attraction of lead ions towards GO causes a shift of 1635 cm^−1^ to 1612 cm^−1^. Also a shift from 1116 cm^−1^ to 1110 cm^−1^ and 1045 cm^−1^ to 1041 cm^−1^ in Si–O stretching vibration is observed.

Furthermore it can be concluded from the blank samples of GO and its comparison with piperazine functionalized GO, that piperazine functionalization has a synergistic effect in increasing adsroption. The EDAX analysis was also performed to evaluate lead ion adsorption. In the EDAX analysis (Fig. 10), it is apparent that the lead ions are far more widespread than nitrogen atoms of piperazine in EDAX mapping.

Accordingly, the cooperative assistance of N–H, OH and COOH groups are responsible for adsorption. The chemical positions of Pb^2+^ ions can be depicted in Fig. 1.

### Optimization by RSM

To achieve maximum lead removal efficiency, the effect of four independent parameters including solution pH (A), Pb^2+^ concentration (B), adsorption dosage (C) and shaking time (D) was performed via RSM design based CCD experiments. According to the obtained data, the quadratic model equation was used to describe the relationship between the removal efficiency (% R) and the effective parameters as well as the interaction of operating parameters. This model is expressed according to Eq. (1) as follows, in terms of coded factors:

3 $$\begin{aligned} \% {\text{ R}} & = \, + {96}.{28 } - { 4}.0{\text{8 A }} - {4}.0{\text{37 B }} + { 6}.{\text{48 C }} + { 12}.{\text{99 D }} \\ & \quad + { 6}.{\text{29 AC }} - { 6}.{\text{86 AD }} - {1}.{\text{94 BD }} + { 8}.{\text{12 CD }} - { 2}0.{3}0{\text{ A}}^{{2}} - { 6}.{\text{41 B}}^{{2}} - {13}.{\text{99 C}}_{{2}} - {9}.{\text{33 D}}^{{2}} . \\ \end{aligned}$$

The ANOVA results for the removal efficiency of Pb(II) is summarized in Table 2. As shown, the F-value was 228.36 and p-value was less than 0.05 (p ˂ 0.0001), therefore implies that the quadratic model was statistically significant. Based on this concept (p ˂ 0.05) factors of A, B, C, D, AC, AD, BD, CD, A^2^, B^2^, C^2^ and D^2^ are significant model terms on removal efficiency of Pb(II) ions. In addition, the lack of fit p-value (0.2516) represented the LOF is not significantly relative to the pure error. The fit of proposed model was evaluated using coefficient of determination R^2^ (0.9938) and adjusted R^2^ (0.9895), which indicate a good relationship between the actual (experimentally observed) values and values predicted by the model, and the predicted-R^2^ (0.9777) showed that the model had a high potential to predict the response^1,4^.

**Table 2:** ANOVA results for Pb(II) removal.

Source	Sum of squares	Df	Mean square	F-value	P-value	
Model	22,574.70	12	1881.23	228.36	< 0.0001	Significant
A-pH	276.76	1	276.76	33.60	< 0.0001	
B-Conc	356.51	1	356.51	43.28	< 0.0001	
C-Ads. Do	1046.76	1	1046.76	127.06	< 0.0001	
D-time	3792.62	1	3792.62	460.38	< 0.0001	
AC	577.20	1	577.20	70.07	< 0.0001	
AD	601.48	1	601.48	73.01	< 0.0001	
BD	42.58	1	42.58	5.17	0.0363	
CD	1038.45	1	1038.45	126.06	< 0.0001	
A^2^	10,940.59	1	10,940.59	1328.05	< 0.0001	
B^2^	1126.77	1	1126.77	136.78	< 0.0001	
C^2^	5024.35	1	5024.35	609.89	< 0.0001	
D^2^	2158.91	1	2158.91	262.06	< 0.0001	
Residual	140.05	17	8.24			
Lack of fit	114.63	12	9.55	1.88	0.2516	Not significant
Pure error	25.42	5	5.08			
Cor total	22,714.75	29				

**Figure 12:**
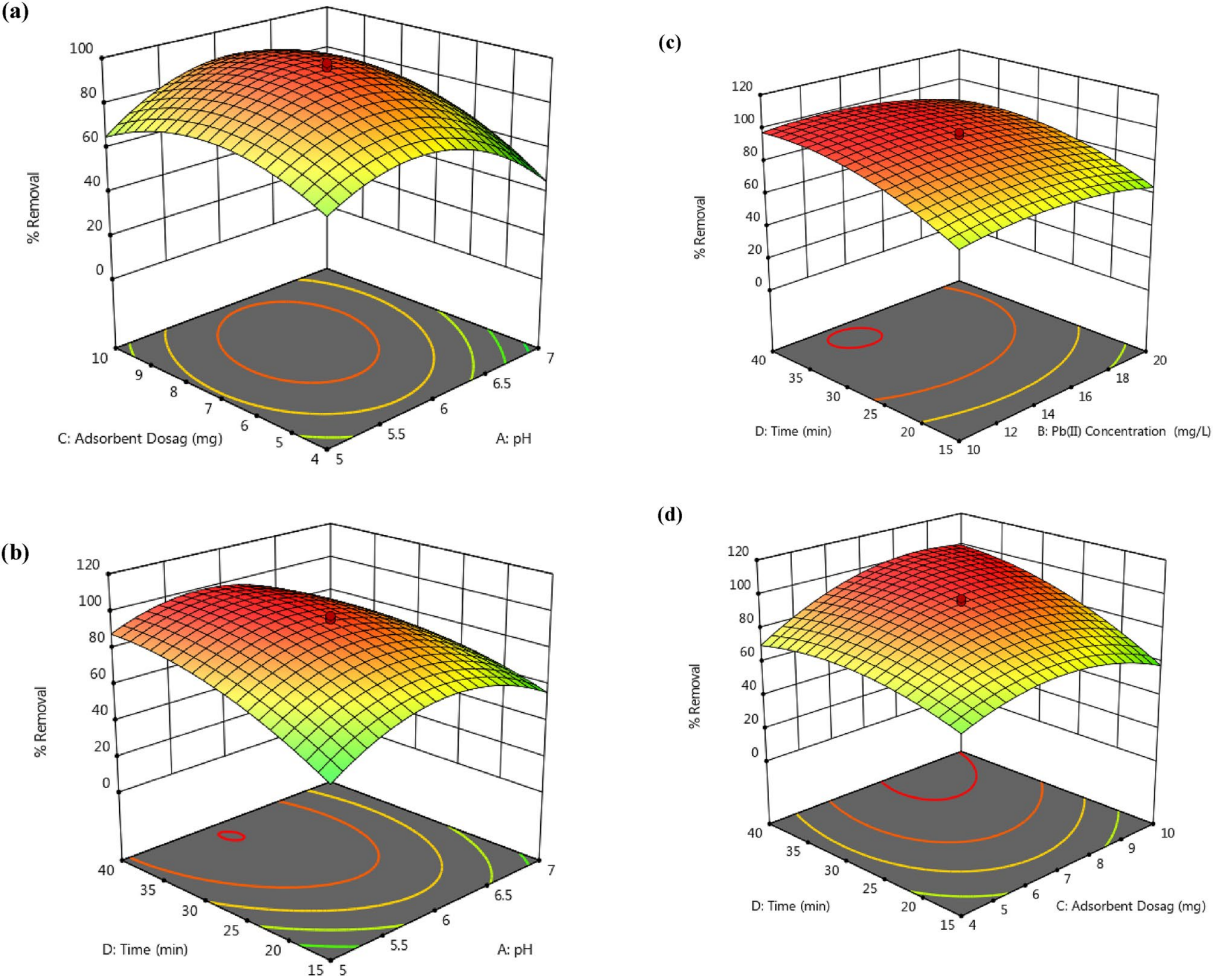
3D response surface plots of Pb(II) adsorption on Pip@MGO nanocomposite.

Three-dimensional (3D) surface plots, which obtained using the CCD design, are given in Fig. 12. By applying these 3D diagrams, the simultaneous effects of independent parameters and their interaction on the removal efficiency can be investigated.

The initial pH value of the media is one of the most significant factors in the adsorption of metal ions^16^. As can be seen from Fig. 12a,b, the maximum removal efficiency of Pb(II) was achieved at pH 6.0. It can be explained that; at low pH values, due to presence of excessive amounts of H^+^ ions in the solution, competition between Pb^2+^ and H^+^ occurs in the active sites on the adsorbent surface and repulsion of Pb^2+^ and protonated amine^39^, as a result the removal efficiency decreases. However, at high pH values, despite the increase in deprotonation of adsorbent surface, Pb(II) ions are converted to hydroxide form and affect the absorption process, eventually reducing the removal efficiency^3,8^. Figure 12a,d shows that increasing the adsorbent dose increases the removal of ions, because it increases the number of adsorbent active sites and enhances the distribution coefficient of Pb(II) ions^4^. It is clear that, the optimum adsorbent dose for Pb^2+^ removal is in the range of 6–8 mg of Pip@MGO nanocomposite. At dose 7 mg, an equilibrium was achieved and on further addition of adsorbent dose percent removal of lead slightly decreased and it is supported by the fact that the active sites get overlapped on the nanoadsorbent surface due to overcrowding^51^.

The removal efficiency of the nanocomposite adsorbent used is affected by the contact time parameter due to the equilibrium nature of lead (II) removal. The results of Fig. 12b,d indicate that the removal efficiency increases with increasing contact time between lead ion and adsorbent, and after 25 min the removal percentage reaches more than 90%, and arrived at nearly constant amount at equilibrium conditions after this time. This variation in adsorption may be due to the relatively high concentration gradient and initially empty surface active sites. Later on at 25 min, the surface active sites get saturated with Pb^2+^ ions and almost constant removal efficiency was acquired^52^.

The initial concentration of the metal ions in the solution is a key driving force in the adsorption of the ions into a solid phase. The effect of initial lead (II) concentration on removal efficiency is shown in Fig. 12c. As it is observed in the removal efficiency decreased on increasing lead (II) concentration. It can be deduced that at higher concentration levels, due to the saturation of the adsorbent active sites with lead ions, the adsorption did not change and the adsorption performance is decreased. Similar results were reported by Moradi et al.^8^, Dehghani et al.^53^ and Bahrami et al.^54^. As a result of experimental investigations, in the concentration range of 14–16 mg L^−1^ of Pb^2+^ ions and in the constant amount of absorbent dosage, the removal process was reached to equilibrium.

The maximum removal efficiency of lead ions was obtained at the optimum value of each independent parameter determined by RSM. The optimal experimental conditions were pH, 6; initial concentration of Pb(II), 15 mg L^−1^; adsorbent dosage, 7 mg; and contact time, 27.5 min. The desirability function value under the optimum conditions was found to be 1, which indicate the accuracy of the RSM divination^55^.

### Adsorption isotherms study

In order to find the adsorption mechanism of lead ions on the Pip@MGO nanocomposite, the experimental data and homogeneity or heterogeneity of adsorbent were analysed by adsorption isothermal models. There are many isotherm models in the literature to describe the analyte adsorption on the adsorbents, however, in the present study, three conventional models including Freundlich, Langmuir and Temikin isotherm equations were used (see supplementary information for more details)^56–59^. For this purpose, in batch mode; 0.01 g of Pip@MGO nanocomposite was added to 10 mL of Pb^2+^ solution with initial concentrations range of 50–600 mg L^−1^, which was stabilized in pH = 6.0, and shacked (200 rpm) for 60 min at 25 °C. Subsequently, the magnetic adsorbent was isolated and the lead equilibrium concentration of each experiment was determined by FAAS, and the experimental data (Supplementary Table S2) were adjusted with the mentioned isotherm models.

Supplementary Figure S1 reveals that upon adsorption equilibrium, the solid phase adsorbed amount of lead ions (*Q*_e_) increased sharply with their aqueous concentration (*C*_e_) in the low *C*_e_ range. Such an increase became less significant at higher *C*_e_ likely due to adsorbent saturation. As the initial Pb(II) aqueous concentration (*C*_0_) increased from 50 to 600 mg L^−1^, *Q*_e_ increased from 64.3 to 525.7 mg g^−1^. Since lead could be classified as a hard Lewis acid, the amine, hydroxyl and carboxyl groups (hard Lewis bases) on the adsorbent probably have a higher affinity for Pb^60^.

To describe the adsorption process, the considered isotherm was determined according to the correlation coefficient of the linear model of the common isotherm equations. Table 3 presents the correlation coefficients (R) and fitting model parameters of Langmuir, Freundlich and Temkin models. As can be seen, the Langmuir model’s correlation coefficient (R = 0.998) is higher than 0.9 compared to other models. In addition, the maximum Langmuir model-determined adsorption value are quite closer to the capacity of lead ions observed experimentally. Since the Langmuir model provided the best fit on Pb^2+^ adsorption by Pip@MGO (Supplementary Fig. S2), the above result indicates that monolayer adsorption of Pb(II) occurred at homogeneous sites with equal energy on nanocomposite adsorbent^60^. Moreover, the maximum adsorption capacity (*q*_*max*_) of Pip@MGO nanocomposite for Pb(II) reached 558.2 mg g^−1^ and it helps the fact that the proposed adsorbent has a high surface area, especially in the nano proportions. The results show that Pip@MGO is an effective adsorbent for removal of Pb^2+^.

**Table 3:** Isotherm parameters for the adsorption of Pb(II) ions onto Pip@MGO nanocomposite.

Isotherm	Parameters	Values of parameters
Langmuir	*R*^2^	0.996
	*K*_L_ (L g^−1^)	0.029
	*q*_m_ (mg g^−1^)	558.2
	*R*_*L*_	0.05–0.41
Freundlich	*R*^2^	0.899
	*K*_F_ (L g^−1^)	1.624
	*n*	0.27
Temkin	*R*^2^	0.893
	*A*_T_ (L g^−1^)	0.38
	*b*_*T*_	20.34
	*B* (J mol^−1^)	121.88

### Adsorption kinetics study

The kinetic study of the adsorption process provides information on the adsorption mechanism and how the Pb(II) ions are transferred from the liquid phase to the solid phase. Therefore, two kinetic models, including pseudo-first-order and pseudo-second-order equations were applied to fit the experimental data (see supplementary information for more details)^61,62^. These experiments were performed by shaking 0.01 g of Pip@MGO nanocomposite in 10 mL solution of 10 mg L^−1^ Pb(II) at pH 6 for 2, 3, 5, 10, 30 and 90 min. The results are presented in Supplementary Table S3.

Table 4 summarizes the regression coefficients and kinetics parameters obtained from data review of the experimental results. According to the results, a good fit is represented by the pseudo-second-order model for the experimental kinetic data in terms of the correlation coefficient (R), because the value obtained for pseudo-second-order (R = 0.999) is greater than R (0.609) for the pseudo-first-order (Supplementary Figs. S3 and S4). The rate-determining step is assumed to be chemisorption in the pseudo-second-order model, including valence forces by exchanging or sharing adsorbate and adsorbent electrons. In addition, the adsorption capacity is proportional to the number of active sites involved on the adsorbent surface^8,63^. Therefore, it can be concluded that the adsorption of Pb^2+^ ions on to Pip@MGO nanocomposite is mostly the chemical reactive adsorption.

**Table 4:** Kinetic parameters for the adsorption of Pb(II) ions with Pip@MGO nanocomposite.

Models	Parameters	Cd(II)
Pseudo-1st-order	*q*_e_ (mg g^−1^)	0.60
	*k*_1_ (min^−1^)	− 0.024
	*R*^2^	0.3707
Pseudo-2nd-order	*q*_e_ (mg g^−1^)	10.41
	*K*_2_ (g mg^−1^ min^−1^)	− 2.052
	*R*^2^	0.999

### Applications and reusability

In order to evaluate the application of the proposed method in the removing lead ions from real samples, three different water and wastewater samples, including Arvand Rud and Bahmanshir rivers, Persian Gulf seawater and petrochemical wastewater samples were investigated. To examine the effect of the sample matrices on the removal process, all samples were spiked by known concentrations of Pb(II) ions at two levels, 5 and 10 mg L^−1^, subsequently the suggested method was performed under optimum conditions. The analysis results of each sample along with the recoveries are presented in Table 5. As can be seen, the removal efficiency of the lead ions in the studied samples were upper 93% which show well suitability of the developed nanocomposite adsorbent for removing of Pb(II) ions in various real samples.

**Table 5:** Removal of Pb(II) from various water and wastewater samples using proposed method.

Sample	Add concentration (mg L^−1^)	Removal (%)
River water (Karun)	5	> 99
	10	> 99
River water (Bahmanshir)	5	> 99
	10	> 99
Wastewater effluent (Abadan Petrochemical Company)	5	> 99
	10	96
Seawater (Persian Gulf)	5	> 99
	10	93

The regeneration cycles of Pip@MGO nanocomposite were carried out using acidic solution. The adsorption/desorption study proved that the adsorbent with nitric acid (1 M) could be reused for four cycles. The results are shown in Supplementary Fig. S5. Based on this figure, the removal efficiency of Pip@MGO nanocomposite in four consecutive adsorption-regeneration cycles is more than 90%. After mentioned cycles, the removal efficiency of lead ions is reduced to less than 90%, and this can be attributed to the deformation of the absorbent material when used again or due to the saturation of the surface of the adsorbent^64,65^. These results show that Pip@MGO nanocomposite can be used multiple times, and the cost of adsorbent preparation can be reduced.

### Comparison to methods in literature

A comparative study of the analytical results of Pb(II) adsorption on the Pip@MGO nanocomposite with other adsorbents in the literature is reported in Table 6. From this table, it is obvious that Pip@MGO nanocomposite has a good adsorption capacity (mg g^−1^) for lead uptake compared to most mentioned adsorbents, while the adsorbent dosage used is less or comparable to other adsorbents.

**Table 6:** Comparative study of adsorption capacity of Pip@MGO nanocomposite with different adsorbents for removal of Pb (II).

Adsorbent	pH	q_m_ (mg g^−1^)	Equilibrium time (min)	Adsorbent Dosage (g/L)	References
Rice husk nanoadsorbent	8	6.1	70	12	^3^
Bentonite enriched-SH groups	5	12	146	1.5	^4^
Fe_3_O_4_@glycidylmethacrylate-acrylamide	6	158.7	10 s	0.2	^8^
GO nanocomposite decorated with NiFe_2_O_4_ nanoparticles	8.5	957	18	–	^45^
EDTA-magnetic GO	4.2	479	20	–	^66^
Thiourea modified magnetic ZnO/nano Celloluse	6.5	554.4	14.5	0.2	^67^
Fe_3_O_4_-EDTA	7.9	112	10	1.1	^68^
Modified red mud	5	551.1	60	1.0	^69^
Fe_3_O_4_/FeMoS_4_/MgAl-LDH	5	190.7	60	3.0	^70^
Amino/thiol bifunctionalized magnetic nanoparticle	5	110.1	3	1.0	^71^
Bifunctionalized GO/MnFe_2_O_4_ magnetic Nanohybrids	5.5	366.4	120	–	^72^
Pip@MGO nanocomposite	6	558.2	27.5	0.7	Present work

## Conclusions

In this present study, we have constructed a new magnetic graphene oxide-functionalized piperazine (Pip@MGO) nanocomposite to remove Pb(II) ions from contaminated waters. The synthesized nanocomposite adsorbent was analyzed by using XRD, FESEM, TEM, EDAX, TGA, VSM and FT-IR techniques. The influence of four key parameters consisting initial pH, adsorbent dosage, initial concentration of lead and contact time on the removal efficiency were evaluated by RSM based on the central composite design (CCD) model. As a result of mathematical optimization, the maximum adsorption efficiency was obtained at pH 6.0, adsorbent dose of 7 mg, initial Pb^2+^ concentration of 15 mg L^−1^ and contact time of 27.5 min.

The isotherm studies indicated that lead adsorption equilibrium data were more appropriate with Langmuir isotherm model. In addition, the obtained equilibrium data were applied to the kinetic equations and found to be consistent with the pseudo-second order model for lead adsorption. The suggested nanocomposite adsorbent represented good adsorption capacity and could be regenerated by nitric acid and reused for up to four adsorption–desorption cycles. According to our findings, the Pip@MGO nanocomposite as an effective adsorbent can be successfully used for the removal of Pb(II) ions from different real samples and changes in the sample matrices do not have a significant effect on the removal efficiency of the proposed methodology.

## Supplementary Information

Supplementary Information.

## Data Availability

All data generated or analysed during this study are included in this published article [and its supplementary information files].
