# Supplementary material for: Effective removal of Pb(II) ions using piperazine-modified magnetic graphene oxide nanocomposite; optimization by response surface methodology
Source: Sci Rep. 2022 Jun 10;12:9658. doi: 10.1038/s41598-022-13959-8 (PMC9187642; doi:10.1038/s41598-022-13959-8)
Supplement: Supplementary file 1 — Supplementary Information. [file 41598_2022_13959_MOESM1_ESM.docx]

**Electronic supplementary material**

**Effective removal of Pb(II) ions using piperazine-modified magnetic graphene oxide nanocomposite; optimization by response surface methodology**

Mousa Alboghbeish ‬^a^, Arash Larki ^a^*, Seyyed Jafar Saghanezhad ^b^

^a^ Department of Marine Chemistry, Faculty of Marine Science, Khorramshahr University of Marine Science and Technology, Khorramshahr, Iran

^b^ ACECR-Production Technology Research Institute, Ahvaz, Iran

***Corresponding author:**

**Arash Larki, E-mail:** [arash_larki@yahoo.com](mailto:arash_larki@yahoo.com) & a.larki@kmsu.ac.ir

**Contents:**

S1. Adsorption study (Pages S2-S3);

S2. Additional 3 tables (Pages S4 & S6);

S3. Additional 5 figures (Pages S7-S11);

References (Page S12);

**S1. Adsorption study**

***Adsorption isotherms***

In the Langmuir model, it is assumed that the adsorption is monolayer and occurs at a homogeneous surface, without interaction between the absorbed materials ^1^. The linearity of the Langmuir model is described by the equation (1) as follows:

$\frac{C_{e}}{q_{e}}=\frac{1}{K_{L}q_{max}}+ \frac{C_{e}}{q_{max}}$ (1)

where, *C*_e_ (mg L^-1^) and *q_e_* (mg g^-1^) are Pb(II) ion concentration and quantity of lead ions adsorbed onto the absorbent surface at equilibrium, *q_max_* (mg g^-1^) is the maximum adsorption capacity and *K_L_* (mL mg^-1^) is the Langmuir adsorption equilibrium constant. *q_max_* and *K_L_* could be calculated with a straight line drawn from of *C_e_*/*q_e_* versus *C_e_*.

The Freundlich isotherm is not accept the capacity of one layer and is based on the assumption of adsorption on heterogeneous surfaces, due to the increase in the amount of analyte adsorbed in the solution ^2^. This model is linear by the equation (2) as follows:

$\log q_{e}=\log k_{f}+\left( \frac{1}{n} \right)\log C_{e}$ (2)

where, *n* and *K_F_* (mg g^-1^ (L mg^-1^)^1/n^) are Freundlich constants, which are related to the heterogeneity factor and adsorption capacity, respectively. These constants can be calculated by plotting of *log q_e_* versus *log C_e_*. The *n* value should be in the range 1 to 10 for favorable adsorption process. The Temkin isotherm model contains a parameter which considers the adsorbent-adsorbate interactions and assumes that the adsorption heat decline linearly with the surface covered between the adsorbates and adsorbent ^3^. The linearity of Temkin model is given by equation (3):

$q_{e}=B\ln K_{T}+\left( \frac{RT}{b} \right)\ln C_{e}$ (3)

where, *B*=*RT*/*b_T_*, *b_T_* (J mol^-1^) is the constant of Temkin that is related to the heat of adsorption, *K_T_* (L g^-1^) is the equilibrium binding constant, *R* and *T* are the gas constant (8.3145 J mol^-1^ K^-1^) and absolute temperature in Kelvin, respectively. The *B* and *K_T_* values can be calculated from slope and intercept of a graph between *q_e_* and *lnC_e_*. The data obtained from the adsorption isotherms study are shown in Table S1.

***Adsorption kinetics***

The pseudo-first-order model was shown in equation (4):

$ln \left( q_{e}-q_{t} \right)=lnq_{e}-K_{1}t$ (4)

where *q*_e_ and *q*_t_ (mg g^-1^) were the amount of adsorbed lead(II) ions at equilibrium and at time *t*; *k*_1_ (min^−1^) was the rate constant of the pseudo-first-order model. Through drawing the plot of *ln*(*q_e_-q_t_*) vs. *t*, *q*_e_ and *k*_1_ were calculated as the slope and intercept.

In addition, equation (5) gave a model of the pseudo-second-order:

$\frac{t}{q_{t}}=\frac{1}{k_{2}q_{e}^{2}}+\frac{1}{q_{e}}$ (5)

where, *k_2_* (g mg^-1^ min^-1^) was the rate constant of pseudo-second-order adsorption. From the slope and intercept of the plots *t*/*q_t_* vs. *t*, the pseudo-second-order rate constant *k*_2_ and *q*_e_ values were acquired ^4,5^. The obtained results from this study was reported in Table S2.

**Table S1**. Experimental results of adsorption tests in removalof Pb(II) ions under CCD.

|  | Factor 1 | Factor 2 | Factor 3 | Factor 4 | Response 1 |
| --- | --- | --- | --- | --- | --- |
| Run | A:pH | B:Pb(II) Concentration | C:Adsorbent Dosag | D:Time | % removal |
|  |  | mg/L | mg | min |  |
| 1 | 6 | 15 | 7 | 2.50 | 31.2 |
| 2 | 7 | 10 | 10 | 40.0 | 73.0 |
| 3 | 7 | 20 | 4 | 15.0 | 29.5 |
| 4 | 6 | 15 | 7 | 52.5 | 88.5 |
| 5 | 6 | 15 | 7 | 27.5 | 96.0 |
| 6 | 6 | 15 | 7 | 27.5 | 91.7 |
| 7 | 5 | 10 | 10 | 40.0 | 81.0 |
| 8 | 6 | 5 | 7 | 27.5 | 76.4 |
| 9 | 5 | 10 | 4 | 40.0 | 65.0 |
| 10 | 6 | 15 | 13 | 27.5 | 56.0 |
| 11 | 4 | 15 | 7 | 27.5 | 21.9 |
| 12 | 7 | 10 | 4 | 15.0 | 35.0 |
| 13 | 6 | 15 | 7 | 27.5 | 96.0 |
| 14 | 6 | 15 | 7 | 27.5 | 97.9 |
| 15 | 6 | 15 | 7 | 27.5 | 93.0 |
| 16 | 7 | 10 | 4 | 40.0 | 34.0 |
| 17 | 5 | 10 | 4 | 15.0 | 44.5 |
| 18 | 8 | 15 | 7 | 27.5 | 9.0 |
| 19 | 6 | 15 | 1 | 27.5 | 26.4 |
| 20 | 5 | 20 | 4 | 40.0 | 52.0 |
| 21 | 7 | 20 | 4 | 40.0 | 22.0 |
| 22 | 5 | 20 | 10 | 15.0 | 25.3 |
| 23 | 5 | 10 | 10 | 15.0 | 21.2 |
| 24 | 5 | 20 | 10 | 40.0 | 70.5 |
| 25 | 6 | 25 | 7 | 27.5 | 63.0 |
| 26 | 7 | 10 | 10 | 15.0 | 45.7 |
| 27 | 5 | 20 | 4 | 15.0 | 34.9 |
| 28 | 6 | 15 | 7 | 27.5 | 94.5 |
| 29 | 7 | 20 | 10 | 40.0 | 62.6 |
| 30 | 7 | 20 | 10 | 15.0 | 36.9 |

**Table S2**. Experimental results for adsorption isotherms.

Experimental conditions; in batch mode; 0.01 g of Pip@MGO nanocomposite was added to 10 mL of Pb^2+^solution with initial concentrations range of 50- 600 mg L^-1^, which was stabilized in pH=6.0, and shacked (200 rpm) for 60 min at 25 °C.

| C_0_  (mg L^-1^) | C_e_  (mg L^-1^) | q_e_(mg/g) | C_e_/q_e_ | ln C_e_ | ln q_e_ | R_L_ |
| --- | --- | --- | --- | --- | --- | --- |
| 50 | 5.0 | 64.2 | 0.08 | 1.61 | 4.16 | 0.41 |
| 100 | 9.1 | 130.0 | 0.07 | 2.20 | 4.87 | 0.26 |
| 150 | 12.0 | 197.1 | 0.06 | 2.48 | 5.28 | 0.19 |
| 200 | 29.0 | 244.3 | 0.12 | 3.37 | 5.50 | 0.15 |
| 250 | 38.3 | 302.9 | 0.13 | 3.64 | 5.71 | 0.12 |
| 300 | 54.2 | 351.1 | 0.15 | 3.99 | 5.86 | 0.10 |
| 450 | 127.0 | 461.4 | 0.28 | 4.84 | 6.13 | 0.07 |
| 600 | 239.4 | 515.7 | 0.46 | 5.48 | 6.25 | 0.05 |

**Table S3**. Experimental data for adsorption kinetics.

Experimental conditions; in batch mode; these experiments were performed by shaking 0.01 g of Pip@MGO nanocomposite in 10 mL solution of 10 mg L^-1^ Pb(II) at pH 6 for 2, 3, 5, 10, 30 and 90 min.

| Time  (min) | q_t_  (mg g^-1^) | q_e_  (mg g^-1^) | t/q_t_ |
| --- | --- | --- | --- |
| 1 | 10.57525 | 10.57525 | 0.09456 |
| 5 | 10.2408 | 10.57525 | 0.488243 |
| 10 | 10.30769 | 10.57525 | 0.970149 |
| 20 | 10.57525 | 10.57525 | 1.891208 |
| 40 | 10.47492 | 10.57525 | 3.818646 |
| 60 | 10.37458 | 10.57525 | 5.783366 |

**Fig. S1.** Adsorption study of Pb(II) ions on Pip@MGO fitted with Langmuir isotherm model.

**Fig. S2.** Langmuir isotherm plot for lead(II) removal by Pip@MGO at 25 °C with pH of 6.0.

**Fig. S3.** Kinetic model of Pb(II) ions adsorption onto the Pip@MGO nanocomposite using pseudo-first-order.

**Fig. S4.** Kinetic model of Pb(II) ions adsorption onto the Pip@MGO nanocomposite using pseudo-second-order.

**Fig. S5.** Reusability study of Pip@MGO nanocomposite at different adsorption-desorption cycles.

1. Dehghani, M. H. *et al.* Statistical modelling of endocrine disrupting compounds adsorption onto activated carbon prepared from wood using CCD-RSM and DE hybrid evolutionary optimization framework: Comparison of linear vs non-linear isotherm and kinetic parameters. *Journal of Molecular Liquids* **302**, 112526, doi:https://doi.org/10.1016/j.molliq.2020.112526 (2020).
2. Bahrami, M., Amiri, M. J. & Bagheri, F. Optimization of the lead removal from aqueous solution using two starch based adsorbents: Design of experiments using response surface methodology (RSM). Journal of Environmental Chemical Engineering 7, 102793, doi:https://doi.org/10.1016/j.jece.2018.11.038 (2019).
3. Arabkhani, P., Javadian, H., Asfaram, A. & Hosseini, S. N. A reusable mesoporous adsorbent for efficient treatment of hazardous triphenylmethane dye wastewater: RSM-CCD optimization and rapid microwave-assisted regeneration. Scientific Reports 11, 22751, doi:10.1038/s41598-021-02213-2 (2021).
4. Tamoradi‬ Babaei, Z., Larki, A. & Ghanemi, K. Application of molybdenum disulfide nanosheets adsorbent for simultaneous preconcentration and determination of Cd(II), Pb(II), Zn(II) and Ni(II) in water samples. Journal of the Iranian Chemical Society, doi:10.1007/s13738-021-02289-7 (2021).
5. Javinezhad, S., Larki, A., Nikpour, Y. & Saghanezhad, S. J. Study on the Application of Cucurbit[6]uril as a Nanoporous Adsorbent for the Removal of 2,4-Dinitrophenol from Wastewaters Analytical and Bioanalytical Chemistry Research 5, 217-228, doi:10.22036/abcr.2018.113797.1180 (2018).
